# Image denoising in acoustic microscopy using block-matching and 4D filter

**DOI:** 10.1038/s41598-023-40301-7

**Published:** 2023-08-14

**Authors:** Shubham Kumar Gupta, Rishant Pal, Azeem Ahmad, Frank Melandsø, Anowarul Habib

**Affiliations:** 1 Department of Chemical Engineering, Indian Institute of Technology, Guwahati, India; 2 Department of Electronics and Electrical Engineering, Indian Institute of Technology, Guwahati, India; 3 Department of Physics and Technology, UiT The Arctic University of Norway, Tromsø, Norway

**Keywords:** Engineering, Materials science, Physics

## Abstract

Scanning acoustic microscopy (SAM) is a label-free imaging technique used in biomedical imaging, non-destructive testing, and material research to visualize surface and sub-surface structures. In ultrasonic imaging, noises in images can reduce contrast, edge and texture details, and resolution, negatively impacting post-processing algorithms. To reduce the noises in the scanned image, we have employed a 4D block-matching (BM4D) filter that can be used to denoise acoustic volumetric signals. BM4D filter utilizes the transform domain filtering technique with hard thresholding and Wiener filtering stages. The proposed algorithm produces the most suitable denoised output compared to other conventional filtering methods (Gaussian filter, median filter, and Wiener filter) when applied to noisy images. The output from the BM4D-filtered images was compared to the noise level with different conventional filters. Filtered images were qualitatively analyzed using metrics such as structural similarity index matrix (SSIM) and peak signal-to-noise ratio (PSNR). The combined qualitative and quantitative analysis demonstrates that the BM4D technique is the most suitable method for denoising acoustic imaging from the SAM. The proposed block matching filter opens a new avenue in the field of acoustic or photoacoustic image denoising, particularly in scenarios with poor signal-to-noise ratios.

## Introduction

In materials science to biology, scanning acoustic microscopes (SAM) have been successfully used to image the surface and interior structures and conduct nondestructive evaluations without causing damage to the material being studied^1^. In addition to its ability to inspect objects, the SAM is also capable of providing ample and precise quantitative information about the inspected items. SAM has a range of capabilities, including the non-invasive micro-structural characterization of materials, the characterization of the mechanical properties of piezoelectric materials on their surfaces and subsurface, structural health monitoring (SHM) of the composite structures, detecting surface defects on polymer circuits, and examining the propagation of anisotropic phonons^2–7^. The technology of SAM holds significant importance in the fiercely competitive and demanding markets of microelectronics and semiconductor industries. It plays a vital role in enhancing mold designs for flip-chip packages and is capable of managing the intricacies involved in miniaturized assemblies, such as chip-scale packages and 3D IC stacks, making it a significant tool in the industry^8, 9^.

The resolution of images generated by SAM at a specific frequency relies on the pixel size or scanning steps in both x and y directions, along with the acoustic beam's spot size. In ultrasonic imaging, images are generated by collecting signals, and the quality of the resulting images can be greatly affected by the presence of noise. Images with noise can cause decreased contrast, loss of edge and texture details, and reduced resolution, which can negatively affect post-processing algorithm performance. Therefore, noise is a critical factor that can contribute to the decline of signal quality in acoustic imaging. Accurate parameter determination from acquired images is reliant on effective image denoising.

The most prevalent and unresolved challenge in ultrasound imaging is the presence of noise from multiple sources, which often leads to significant degradation of image quality. Consequently, the presence of noise becomes highly limiting in sensitive applications where acoustic contrast plays a crucial role. Due to various factors such as the environment, electronic noise, transmission cable, and others, images are inevitably subject to noise during acquisition, compression, and transmission, resulting in distortion and loss of image information. These factors make images vulnerable to the manifestation of random noise during data acquisition. Denoising techniques can be classified into two main categories: spatial domain methods and transform domain methods. Spatial filters can be further divided into linear and non-linear filters, and they use low-pass filtering on the pixel values of an image since noise tends to occupy higher regions in the frequency spectrum^10^. Spatial filters tend to reduce noise to a certain extent, but they often lead to blurring of the image. In contrast, the transform domain provides various signal processing techniques, such as wavelet decomposition and empirical mode decomposition (EMD), to tackle this problem^11^. Additionally, methods like principal component analysis (PCA) and singular value decomposition (SVD) can be used for signal reconstruction and restoration^12, 13^. Wang et al. utilized a hybrid method that combined wave packet decomposition and EMD to denoise signals and subsequently classified various engine faults using Support Vector Machine (SVM)^14^. In a separate study, Fan et al. presented a denoising algorithm based on principal component analysis (PCA) that was demonstrated using simulated data with varying levels of noise^15^. Huan et al. introduced a method called C-PCASVD, which combines principal component analysis (PCA) and singular value decomposition (SVD) to identify the singular values of interference^16^. This technique enables an optimal balance between the denoised free induction decay (FID) and the efficiency of noise reduction.

In recent times, artificial intelligence (AI) techniques, and more specifically deep learning (DL) approaches, have demonstrated state-of-the-art performance for many denoising algorithms^17–21^. Zhang et al. introduced DnCNN, a popular deep convolutional neural network (CNN) for image denoising^22^. Other significant contributions in denoising include deep belief networks (DBN)^23^, stacked auto-encoders (SAE)^24^, CNN^25^, and non-local neural networks^26^. CNN-based architectures often excel in handling additive white Gaussian noise (AWGN) but may struggle with other types of noise. Additionally, deep learning models for ultrasound image denoising often require large training datasets, but denoising autoencoders with convolutional layers have shown promising results even with smaller sample sets^24^. Another approach to de-speckling ultrasound images is using PCANet^27^, which has been adapted to include the classical concept of non-local means (NLM)^28^. The Variational Denoising Network (VDN) is a Bayesian-based denoising model that integrates noise estimation with image denoising^29, 30^. However, deep learning-based image denoising faces a significant challenge of requiring a large amount of data, which can be difficult to obtain in acoustic imaging except for medical sonography. Moreover, the training and validation process typically involves specific images, making it a sample-specific, computationally expensive, and time-consuming approach.

Transform domain filtering methods can be categorized into data-adaptive transform, non-data-adaptive transform, block-matching and 3D/4D filtering (BM3D and BM4D) filters^29–31^. Transform domain filtering methods optimize denoising by first transforming the noisy image into a different domain. This approach leverages specific characteristics and noise properties of the transformed image for effective denoising. In the case of the BM4D filter, it performs nonlocal similarity characterizing on a set of consecutive images by utilizing a technique called grouping and collaborative filtering. In the grouping stage, groups are formed as 3-D arrays of mutually similar blocks extracted from the set of consecutive image frames. Several blocks from the same image may be included in a group, naturally taking advantage of the nonlocal similarity. Nonetheless, the majority of blocks that are mutually related may often be identified along the temporal dimension. Then collaborative filtering generates unique estimates for each group by compressing the transform domain of the individual group. When the prevalence of comparable groups next to one another and the high local correlation of the image data are confirmed, each individual group shows correlation in measurements across all dimensions. Applying a decorrelating transformation to the grouped data will result in a sparse representation of the true signal. In contrast to BM3D filtering, which can introduce artifacts and show limited effectiveness in denoising specific image regions, BM4D groups spatiotemporal volumes based on their similarities. In BM4D, the groups are 4D stacks of 3D volumes, and collaborative filtering is performed using a separable 4D spatiotemporal transform. Due to the extreme sparsity of the 4D group spectrum, the noise reduction achieved in BM4D is more successful compared to BM3D. This makes BM4D particularly effective in reducing noise in regions where visual attention is mainly focused. This paper introduces a novel image-denoising method named BM4D, which utilizes block matching and four-dimensional filtering in the three-dimensional transform domain. The 3D transform offers superior mathematical properties compared to commonly used wavelet or contourlet transforms, effectively capturing anisotropic image properties at various scales and directions. By extending BM3D into four 4D spaces, the method significantly enhances edge and texture details in the images. We applied the 4D block matching filter to low-amplitude signal scans with low signal-to-noise ratio and compared its performance with other denoising filters (Gaussian, median, and Wiener filters) using peak signal-to-noise ratio (PSNR) and structural similarity index measure (SSIM) evaluation.

## Theory

### BM4D

In the BM4D algorithm, noisy volumetric data is considered as observation z : X → ℝ of the form

1 $$z(x) = y(x) + \eta (x),\quad x \in X$$

Here, in this equation, y is the original, unknown, and volumetric signal, x is a 3-D coordinate belonging to the signal domain $X \subset {\mathbb{Z}}^{3},$ and $\eta (\cdot ) \sim N(0, {\sigma }^{2})$ is independent and identically distributed (i.i.d.) Gaussian noise with zero means and known standard deviation σ.

BM4D works in two cascading stages, which are a hard-thresholding and a Wiener-filtering stage.

#### Hard-thresholding stage

At the Hard-Thresholding Stage, the four-dimensional groups are created by stacking up noisy, three-dimensional cubes identical to ${C}_{{x}_{R}}^{z}$ along a fourth additional dimension. Here, ${C}_{{x}_{R}}^{z}$ denotes a cube of ${L}^{3}$ where $L \in {\mathbb{N}}$, extracted from noisy observation z at the 3D coordinate ${x}_{R} \in X$. More specifically, the photometric distance is used to calculate how similar two cubes are, which is defined by,

2 $$d\left({C}_{{x}_{i}}^{z} ,{C}_{{x}_{j}}^{z}\right)= \frac{{\parallel {C}_{{x}_{i}}^{z} - {C}_{{x}_{j}}^{z}\parallel }_{2}^{2}}{{L}^{3}}$$

where ${|| \cdot ||}_{2}^{2}$ denotes the sum of squared differences between corresponding intensities of the two input cubes, and the denominator ${L}^{3}$ serves as a normalization factor. No prefiltering is performed before cube matching, so the similarity of noise observations can be directly tested. In the grouping step, cubes similar to each other are extracted from z and combined to form a group for each cube ${C}_{{x}_{R}}^{z}$. If the distance between two cubes was not larger than the predefined threshold ${\tau }_{match}^{ht}$, the two cubes are considered to be similar. Similarly, to ${C}_{xR}^{z}$, we here firstly define a set that contains the indexes for the cubes as follows,

3 $${S}_{{x}_{R}}^{z}=\left\{ {x}_{i} \in X : d\left({C}_{{x}_{R}}^{z} , {C}_{{x}_{i}}^{z} \right)\le {\tau }_{match}^{ht}\right\}$$

Then, a four-dimensional group is built by the above formula (being ${\coprod }$ the disjoint union operation):

4 $${G}_{{S}_{{x}_{R}}^{z}}^{z}= {\coprod }_{{x}_{i}\in {S}_{{x}_{R}}^{z} }{C}_{{x}_{i}}^{z}$$

where the reference cube (represented by R) matches a set of similar cubes located in the 3D data. Particularly, the coordinates ${x}_{R}$ and ${x}_{i}$ correspond to the tail and head of the arrow connecting the cubes in formula (4), respectively.

In the collaborative filtering step, a joint four-dimensional transformation ${T}_{4D}^{ht}$ was applied to each dimension of Eq. (5), respectively. Then, by a hard threshold operator ${\gamma }^{ht}$ with the threshold $\sigma {\lambda }_{4D}$, the obtained four-dimensional group spectrum is

5 $${\gamma }^{ht} {( T}_{4D}^{ht} {(G}_{{S}_{{x}_{R}}^{z}}^{z}))$$

Note that since the distance from any cube to itself is always 0, according to the definition of formula (4), each formula (5) must contain at least its reference cube. Representing the filter group, it is transformed into the following form:

6 $${( T}_{4D}^{ht-1}({\gamma }^{ht} {( T}_{4D}^{ht} {(G}_{{S}_{{x}_{R}}^{z}}^{z})))= {\widehat{G}}_{{S}_{{x}_{R}}^{z}}^{y}= {\coprod }_{{x}_{i}\in {S}_{{x}_{R}}^{z} }{\widehat{C}}_{{x}_{i}}^{y}$$

For each unknown volume data y, the estimated ${\widehat{C}}_{{x}_{i}}^{y}$ of the original ${C}_{{x}_{i}}^{y}$ was extracted separately. Formula (6) was an overcomplete representation of the denoising data because cubes in different groups, as well as cubes within the same group, are likely to overlap; thus, within the overlapping regions, different cubes provide multiple, and in general different, estimates for the same voxel. In the aggregation step, such redundancy is exploited through an adaptive convex combination to produce the basic volumetric estimate

7 $${\widehat{y}}^{ht} = {\sum }_{{x}_{R\in X}} \frac{({\sum }_{{x}_{i\in {S}_{{x}_{R}}^{z}}} {\omega }_{xR}^{ht} {\widehat{C}}_{{x}_{i}}^{y})}{({\sum }_{{x}_{i\in {S}_{{x}_{R}}^{z}}} {\omega }_{xR}^{ht} {\chi }_{{x}_{i}})}$$

where ${\omega }_{xR}^{ht}$ are group-dependent weights, ${\chi }_{{x}_{i}} : X \to \{0, 1\}$ is the characteristic (indicator) function of the domain of ${\widehat{C}}_{{x}_{i}}^{y}$ (i.e., ${\chi }_{{x}_{i}}$ = 1 over the coordinates of the voxels of ${\widehat{C}}_{{x}_{i}}^{y}$ and ${\chi }_{{x}_{i}}$ = 0 elsewhere), and every ${\widehat{C}}_{{x}_{i}}^{y}$ is assumed to be zero-padded outside its domain. Note that, whereas in BM3D, a 2-D Kaiser window of the same size as the blocks is used to alleviate blocking artefacts in the aggregated estimate, in the proposed BM4D, we do not perform such windowing because of the small size of the cubes. The weights in Eq. (7) are defined as

8 $${\omega }_{xR}^{ht}= \frac{1}{{{\sigma }^{2}N}_{{x}_{R}}^{ht}}$$

where σ is the standard deviation of the noise in z and ${N}_{{x}_{R}}^{ht}$ denotes the number of non-zero coefficients in Eq. (5). Since the DC (discrete cosine) coefficient is always retained after thresholding, i.e., ${N}_{{x}_{R}}^{ht}$ ≥ 1, the denominator of Eq. (8) is never zero. The number ${N}_{{x}_{R}}^{ht}$ has a double interpretation: on one hand, it measures the sparsity of the thresholded spectrum (Eq. 5), and on the other, it approximates the total residual noise variance of the group estimate (Eq. 6). As a result, groups with higher correlation levels are rewarded with bigger weights, whilst groups with higher residual noise levels are penalized with smaller weights.

#### Wiener-filtering stage

Following the preceding step, the BM4D algorithm employs Wiener filtering, which is a well-established and widely used adaptive filter in signal processing^32–35^. The Wiener filter is known for its simplicity, stability, and speed, and has been demonstrated to be an optimal filter in various signal-processing applications. Specifically, in BM4D, the Wiener filter is utilized to remove noise from each 3D block of wavelet coefficients, allowing the algorithm to estimate the original, clean signal from the noisy input.

9 $${P}_{wiener} = {\mu }_{m} +\frac{max\left(0, {\sigma }_{m}^{2} - {v}^{2} \right) }{max\left(\sigma {m}^{2}, {v}^{2}\right)}\left({P}_{raw} - {\mu }_{m}\right)$$

where ${P}_{raw}$ represents the raw data in the detector domain. Denoting ${\mu }_{m}$ and ${\sigma }_{m}^{2}$ as local mean and local variance of ${P}_{raw}$ respectively, ${v}^{2}$ as the mean of local variance ${\sigma }_{m}^{2}$. ${P}_{wiener}$ is the denoising result after wiener filtering.

Following the above steps, the BM4D filtering process is performed on noisy 3D domain data, and the final result is obtained after the Wiener filtering stage.

### Flowchart of the algorithm

**Figure 1:**
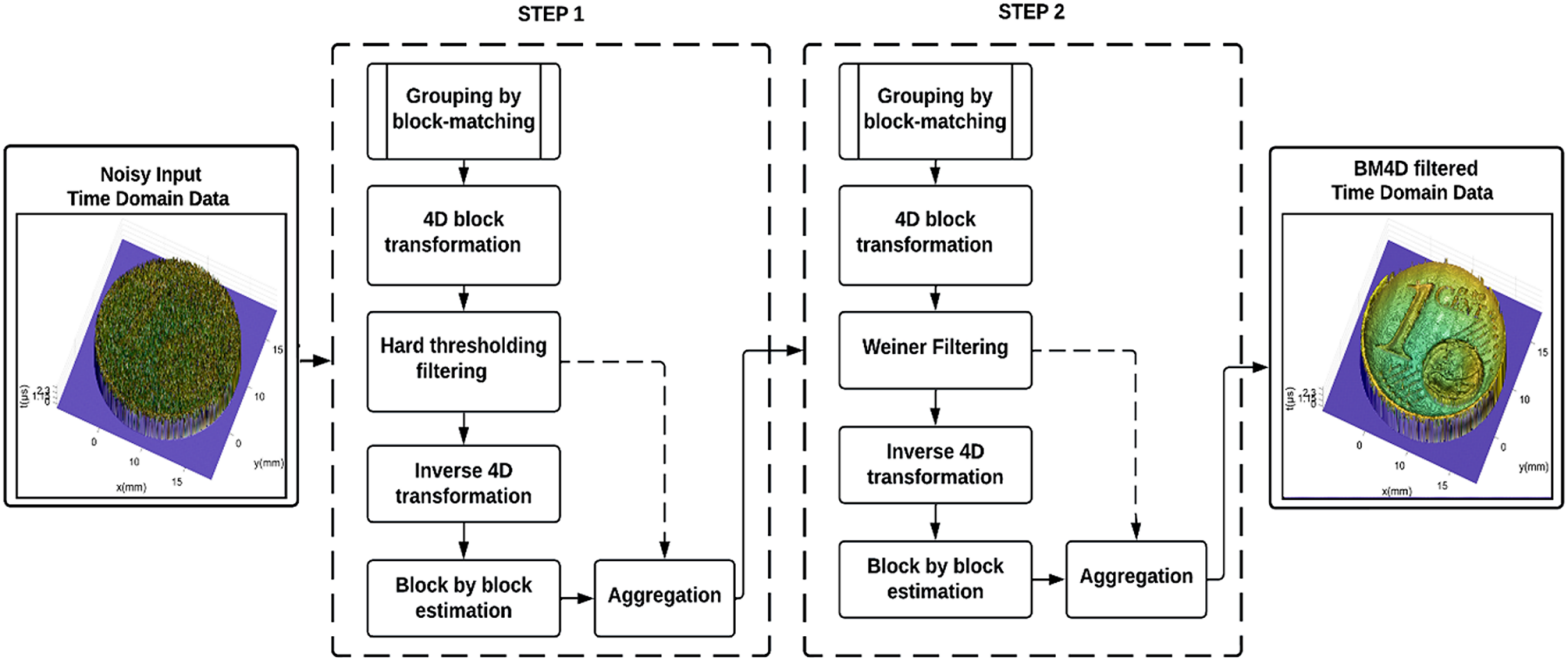
This figure illustrates the steps involved in the 4D block matching filter that is performed over noisy data, here low amplitude signal 0.24 V signal has been taken as input data and the right-hand side output represents the BM4D filtered result.

In this paper, the BM4D algorithm is used to denoise the acquired images through the experimental setup of SAM technique. The applied algorithm is further divided into two steps. In the first step, the noisy 3D domain data is first grouped by block matching algorithm and step 1 filtered output is obtained by hard threshold filtering followed by estimation and aggregation. After that, in the 2nd step, we applied the wiener filtering on the output of step 1. The final denoised output image is then collected at the end of step 2 after aggregating the filtered blocks in the 4D domain. A flowchart of the working process of the applied BM4D algorithm is shown in Fig. 1.

## Experimental setup

**Figure 2:**
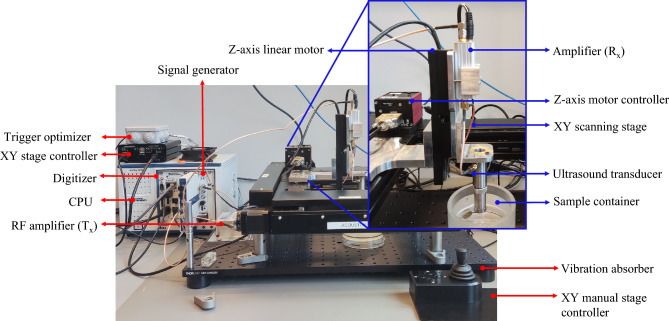
This figure displays a labelled image of the SAM used for image acquisition, showcasing all the essential components that make up a SAM in the experimental setup.

Figure 2 shows a labelled image of SAM, which is used to acquire images of the sample. SAM uses reflection and transmission modes to create images that reveal different features of the sample. An image (Fig. 2) of SAM has been annotated and is used for image acquisition. Further details regarding the working principles of these modes can be found elsewhere. In this article, we have focused on using the reflection mode to scan the samples^36^. A concave spherical sapphire lens rod is frequently used to focus acoustic energy through a coupling medium (in this case, water), and ultrasonic signals are generated from a signal generator and sent to the sample. After the waves bounce back the signals from the sample, they are detected and converted into a digital signal, which is called an A-scan or amplitude scan. To create a C-scan image of the sample, this procedure is performed at different locations in the XY plane. Another way to visualize a C-scan is as the combination of A-scans in two dimensions.

A LabView program controlled a custom-built SAM, as depicted in Fig. 2, which incorporated a Standa high-precision scanning stage (8MTF-200-Motorized XY Microscope Stage) for data collected during the experiment. In a previous study by the same group, a comparable experimental arrangement was used to account for inclined samples^37^. The acoustic imaging capabilities were enabled using National Instruments' PXIe FPGA modules and FlexRIO hardware, which were housed in a PXIe chassis (PXIe-1082) containing an arbitrary waveform generator (AT-1212). The transducer was excited using Mexican hat signals and boosted using an RF amplifier (AMP018032-T) to amplify the ultrasonic signals. The acoustical reflections produced by the sample surface were amplified using a custom-designed amplifier, and then, they were further amplified with a custom-designed pre-amplifier and digitized using a 12-bit high-speed (1.6 GS/s) digitizer (NI-5772). For ground truth, a 50 MHz focused transducer produced by Olympus was utilized, which had a 6.35 mm aperture and a 12 mm focal length.

For this paper, a series of experimental tests were conducted using a custom desised SAM in conjunction with a scanning stage. This stage was utilized to create a custom-designed ultrasonic scanning platform that could be controlled using LabVIEW software, as described in detail in reference^38^. To implement the ultrasonic functionality of the microscope, we. During the experiments, the microscope was focused on a point that was approximately in the middle of the sample. To minimize scanning time and turbulence caused by quick transducer motions, a serpentine mode was employed for stage scanning. The entire experimental setup is illustrated in Fig. 2. In the beginning on the experiment a ground truth or reference scan was performed with 0.65 V_pp_ ( maximum 1 V_pp_) from the signal generator. Later on, 2 other experiments were performed with 0.24 and 0.25 V_pp_, respectively. These 2 exepriments were considered as nosiy scanned data.

### Analysis of the output from SAM

**Figure 3:**
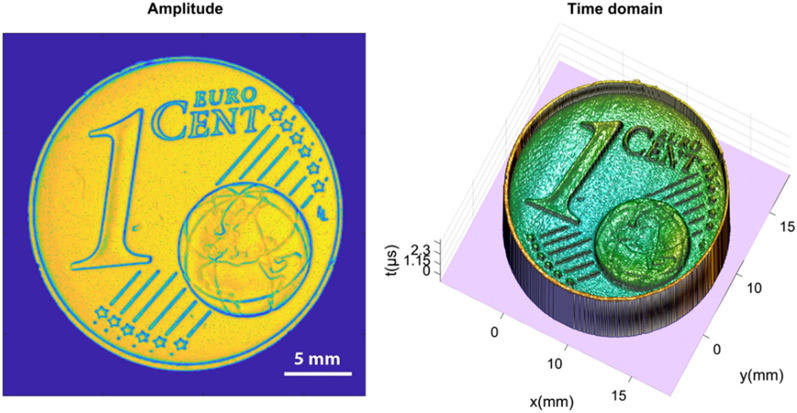
The figure presented in this context displays two visual representations of the ground truth data associated with the Euro 1-cent coin. Left imgae represents an amplitude image and right image representing a time domain mesh plot, which presents a three-dimensional visualization of the ground truth data.

For this experiment, a standard Euro 1-cent coin was scanned as a reference sample at various amplitudes. The scans obtained from the experiment under general conditions contain noises which are introduced due to several reasons like environmental parameters, instrument and measurement error and other reasons. Later we tried to remove noises from these scans using the proposed algorithm. The ground truth or almost noise-free scan is also obtained from the experiment where all the reading and scan parameters are taken in almost ideal conditions to reduce noise parameters. Figure 3 shows the ground truth or the reference image which is finally used to compare with the results obtained via various denoising filters.

### Qualitative analysis of denoised images

In this paper, we explored different denoising filters to enhance the accuracy and obtain noise-free results for the time domain signals. Among the filters tested were the Gaussian filter, median filter, Wiener filter, and 4D block matching (BM4D) filter. Notably, the BM4D filter demonstrated promising outcomes, prompting us to further improve the approach by applying the 3D block matching filter to the denoised signal.

To evaluate the filters' effectiveness in handling low-amplitude data, we used two sets of data with amplitudes of 0.24V_pp_ and 0.25V_pp_, respectively. Through thorough analysis and comparison, we determined that the BM4D filter, along with the supplementary 3D block matching filter, provided the most favorable denoising results, making it an excellent choice for enhancing the quality of low-amplitude data in our study.

**Figure 4:**
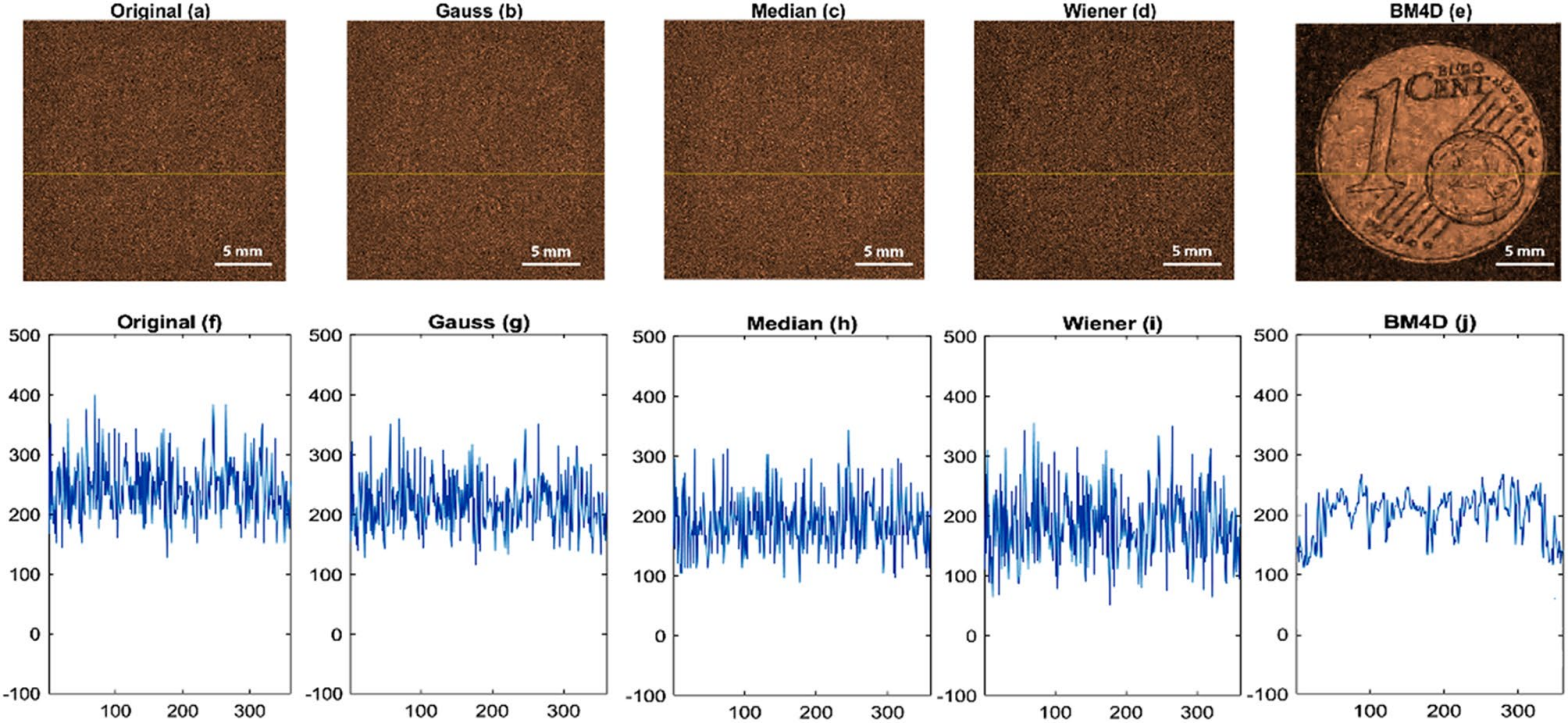
The figure illustrates the utilization of multiple denoising filters, including Gaussian, Median, Wiener filters, and BM4D on low-amplitude signal data with an amplitude of 0.24V_pp._ Each filter's denoised outputs are presented, facilitating a comparison of their noise reduction capabilities and signal clarity enhancement. This analysis enables the evaluation of how effectively these filters handle denoising for signals with low amplitudes.

In Fig. 4, we have presented the results of applying several denoising filters to low-amplitude signal data with an amplitude of 0.24V_pp_, in order to assess their effectiveness in improving the quality of the output. Specifically, we have included an amplitude image and the corresponding line profile at the Y = 201 (shown by the yellow line) of the corresponding images, for each of the following denoising filters: (a) the original noisy data, (b) the amplitude image after applying a Gaussian filter to the time domain signals of the noisy data, (c) the amplitude image after applying a median filter to the time domain signals of the noisy data, (d) the amplitude image after applying a Wiener filter to the time domain signals of the noisy data, and (e) the amplitude image after applying a 4D block matching filter to the time domain signals of the noisy data.

Figure 4 provides clear evidence that the BM4D filter outperforms other filters in denoising the original noisy image. The image quality significantly improves with the BM4D filter, surpassing the results obtained by other filters. The line profile analysis further confirms that the BM4D filter effectively reduces total noise in the image. Moreover, the BM4D filter manages to retain the image structure while effectively removing most of the noise, demonstrating its superior denoising capabilities. This makes the BM4D filter a preferred choice for achieving high-quality denoising results in our study.

**Figure 5:**
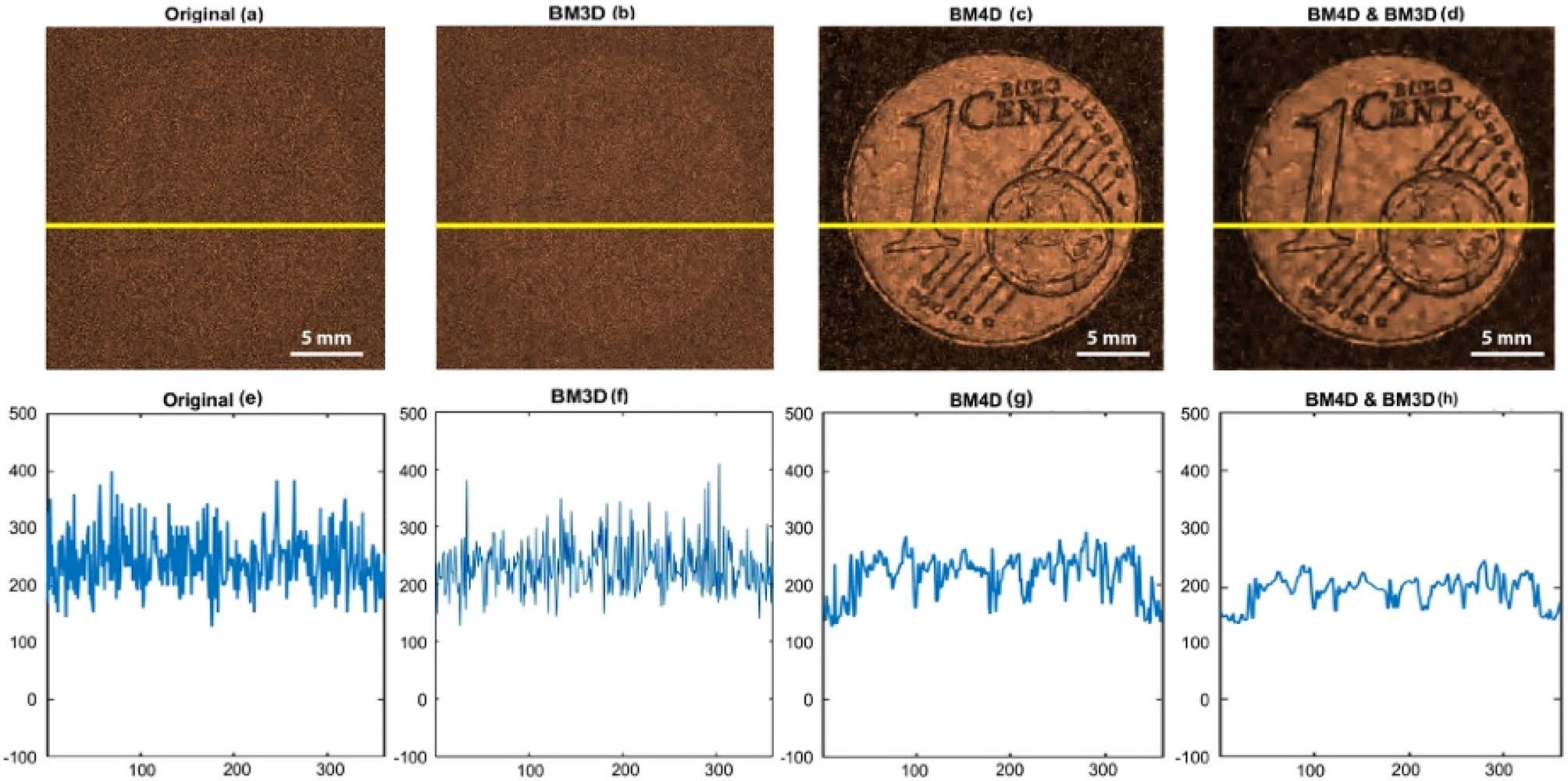
The figure illustrates the application of three denoising filters, namely BM3D, BM4D, and a combined approach of BM4D and BM3D, to low-amplitude signal data with an amplitude of 0.24V_pp_. The denoised outputs obtained from each filter are displayed to represent their performance in reducing noise and enhancing the clarity of the signal. This analysis allows for a comprehensive comparison of the effectiveness of these filters in handling low-amplitude signal denoising.

In Fig. 5, we have provided an assessment of the effectiveness of 4D and 3D block-matching filters in improving the quality of the output obtained from processing low-amplitude signal data with an amplitude of 0.24V_pp_. Specifically, the figure includes (a) the original noisy data, (b) the amplitude image after applying a 4D block-matching (BM4D) filter to the time domain signals of the noisy data, and (c) the amplitude image after applying a 3D block-matching (BM3D) filter to the image obtained from filtering the 4D block matching filtered data. Additionally, the corresponding line profile at the Y = 201 (shown by the yellow line) of the images is included in the figure. After assessing the images in Fig. 5 qualitatively, we can observe that the BM4D filter does remove the noises from the original image effectively. But applying the BM3D filter on the denoise image acquired from the BM4D algorithm substantially reduces the noises while also retaining image structure and overall quality. This assessment can also be verified from the line profile which gives us a general idea of the overall performance of the combined BM4D and BM3D filter.

**Figure 6:**
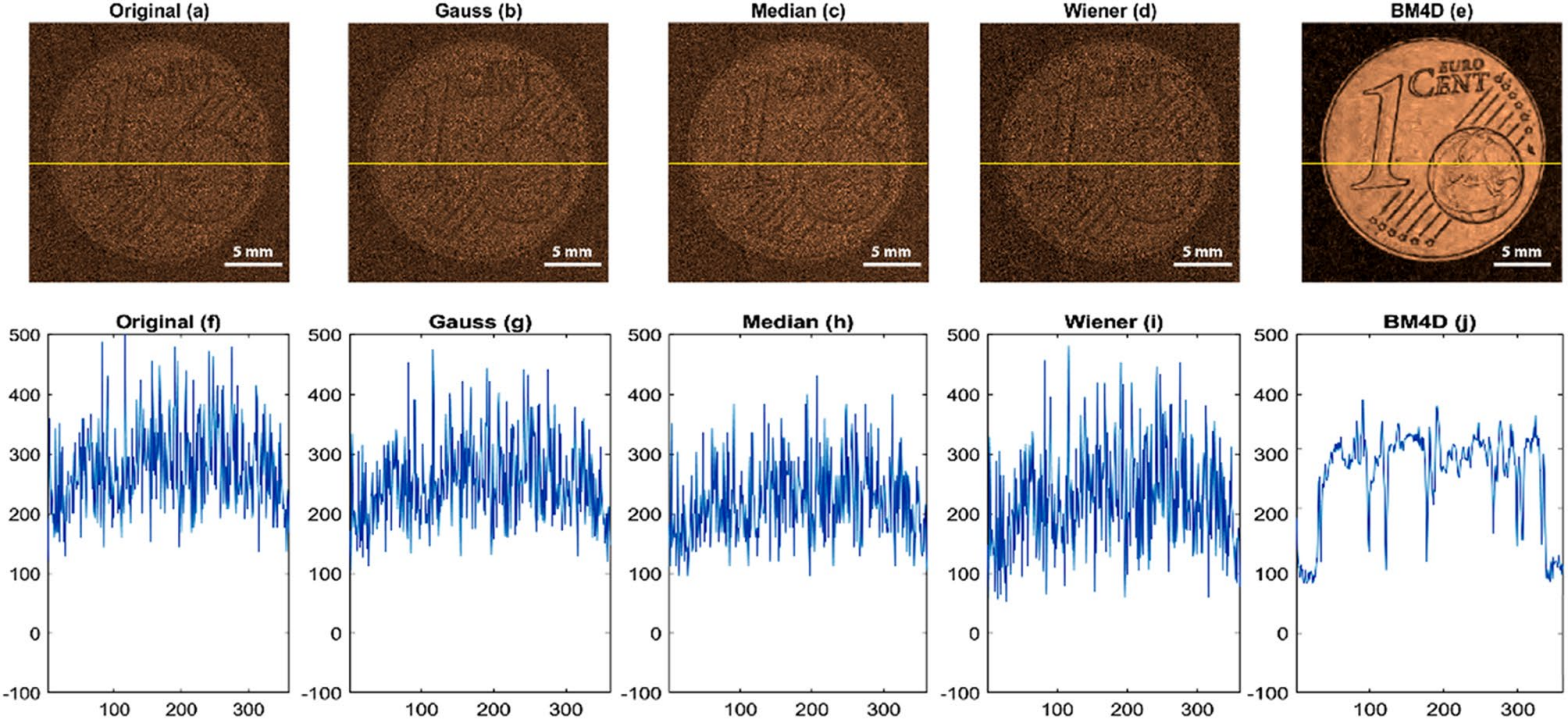
The figure demonstrates the application of various denoising filters to low-amplitude signal data with an amplitude of 0.25V_pp_. The denoised outputs obtained from each filter (Gaussian, Median, Weiner filter, BM4D) are displayed, enabling a comparison of their performance in reducing noise and enhancing the clarity of the signal. This analysis allows us to evaluate the effectiveness of the different filters in handling denoising for signals with low amplitudes.

**Figure 7:**
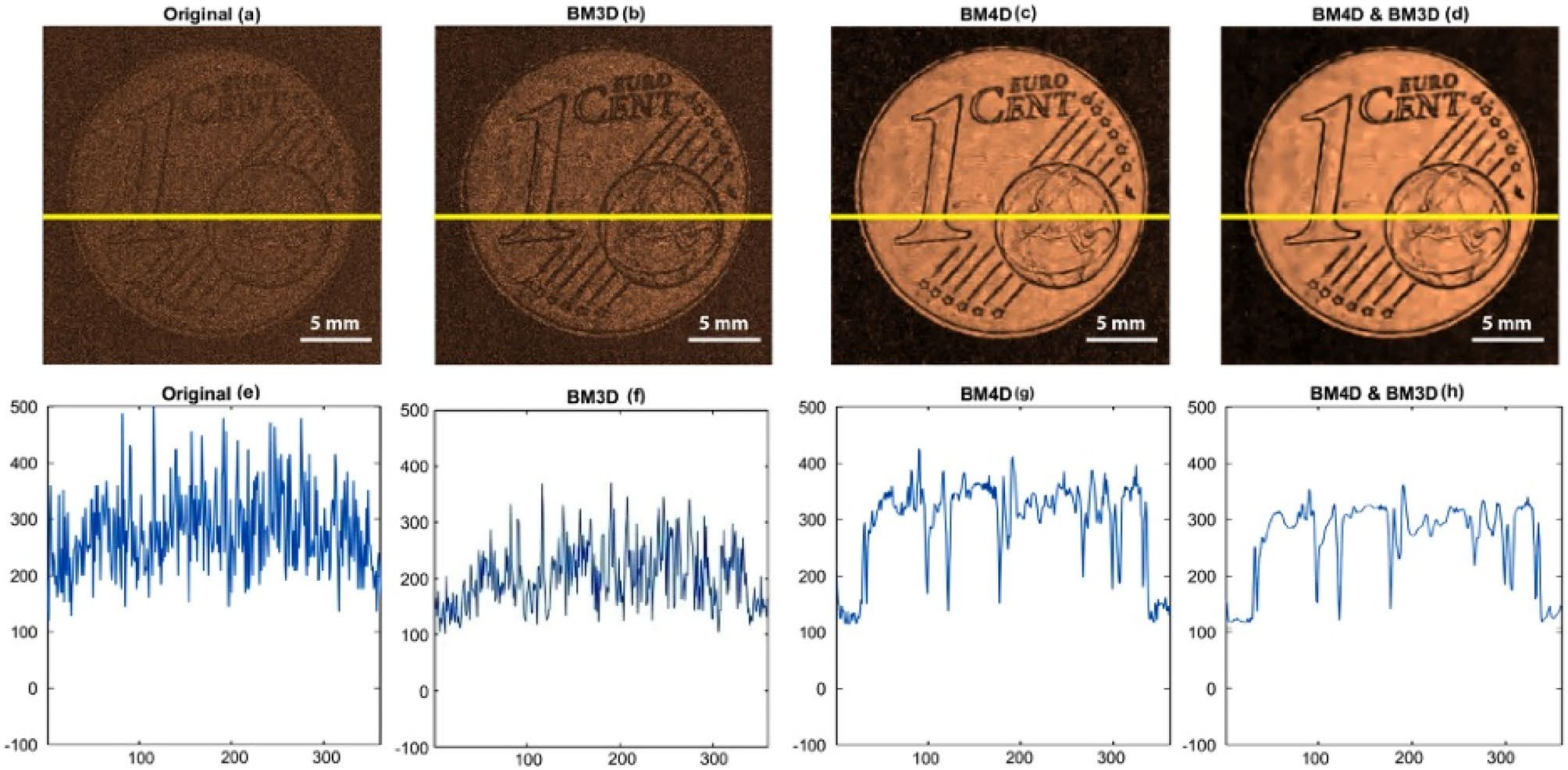
In the presented figure, the denoising process is illustrated for low-amplitude signal data with an amplitude of 0.25Vpp. Three different denoising filters, namely BM3D, BM4D, and a combined BM4D and BM3D filter, are applied to the input signal.

Similarly in Fig. 6, we have presented the results of applying several denoising filters to low amplitude signal data with an amplitude of 0.25V_pp_, in order to assess their effectiveness in improving the quality of the output. Specifically, we have included an amplitude image and the corresponding line profile at the Y = 201 (shown by the yellow line) of the corresponding images, for each of the following denoising filters: (a) the original noisy data, (b) the amplitude image after applying a Gaussian filter to the time domain signals of the noisy data, (c) the amplitude image after applying a median filter to the time domain signals of the noisy data, (d) the amplitude image after applying a Wiener filter to the time domain signals of the noisy data, and (e) the amplitude image after applying a 4D block matching filter to the time domain signals of the noisy data. Furthermore, Fig. 7f–j includes the corresponding line profiles at Y = 201, indicated by the yellow line, for the images.

In Fig. 7, we have provided an assessment of the effectiveness of 4D and 3D block-matching filters in improving the quality of the output obtained from processing low amplitude signal data with an amplitude of 0.25V_pp_. Specifically, the figure includes (a) the original noisy data, (b) the amplitude image after applying a 3D block-matching filter to the time domain signals of the noisy data, (c) the amplitude image is obtained by applying a 4D block-matching filter to the time domain signals of the noisy data, and (d) the amplitude image after applying a 3D block-matching filter to the image obtained from filtering the 4D block matching filtered data. Additionally, the corresponding line profiles at the Y = 201 (shown by the yellow line) of the images are included in the Fig. 7e–h.

The denoising results clearly demonstrate that the BM4D filter is highly effective in reducing noise and enhancing the quality of low-amplitude signal data with an amplitude of 0.25Vpp. Compared to other conventional filters like Gaussian, median, and Wiener filters, the BM4D filter shows the best performance in terms of noise reduction and preserving image structures. Moreover, the combination of BM4D and BM3D filters yields even better results, further enhancing the clarity of the denoised output.

**Figure 8:**
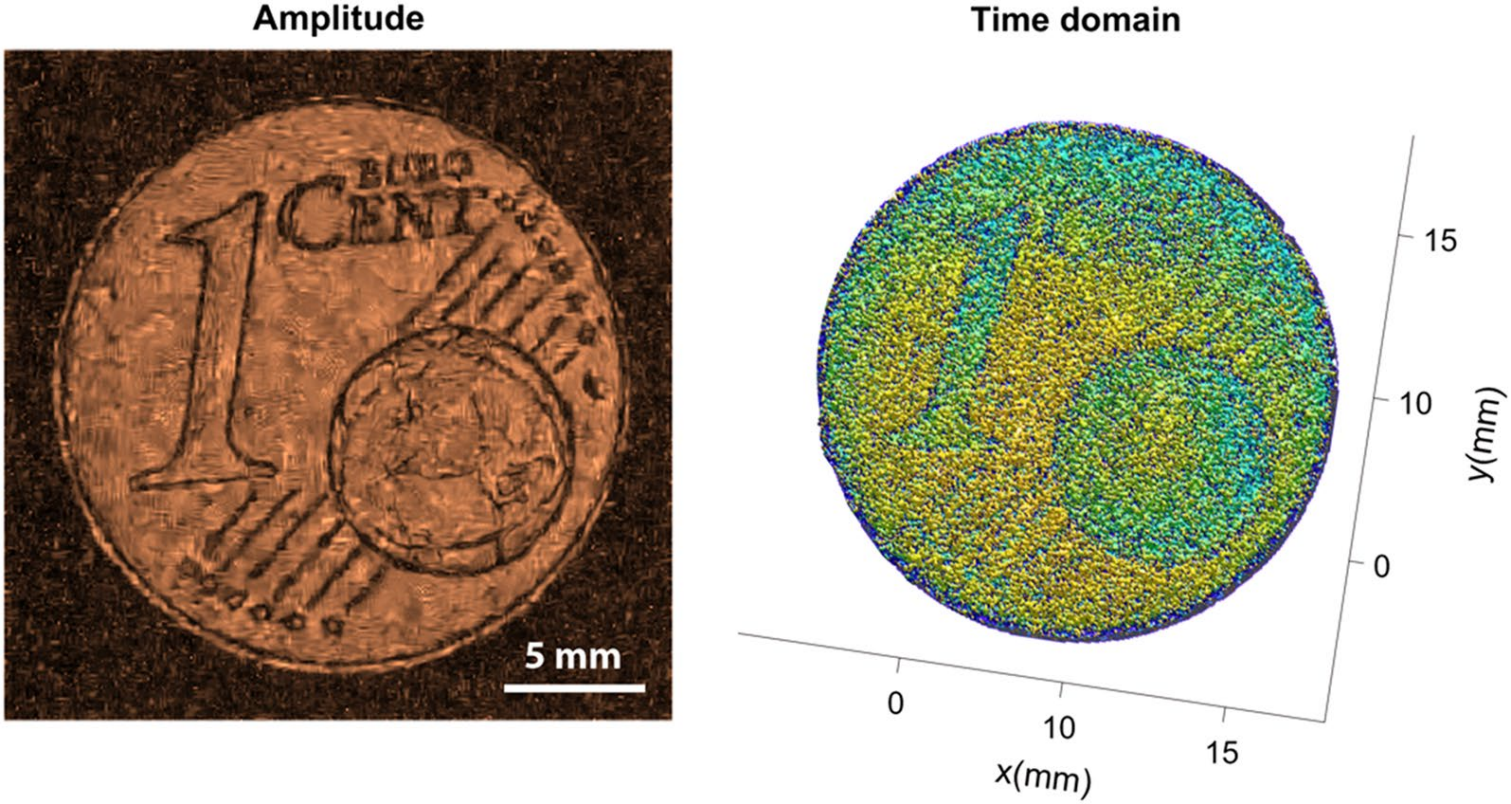
The figure illustrates the results of applying the BM4D filter to low-amplitude signals with an amplitude of 0.24V_pp_. It presents both the amplitude image (left) and the corresponding time domain image (right) obtained after the denoising process using the BM4D filter.

**Figure 9:**
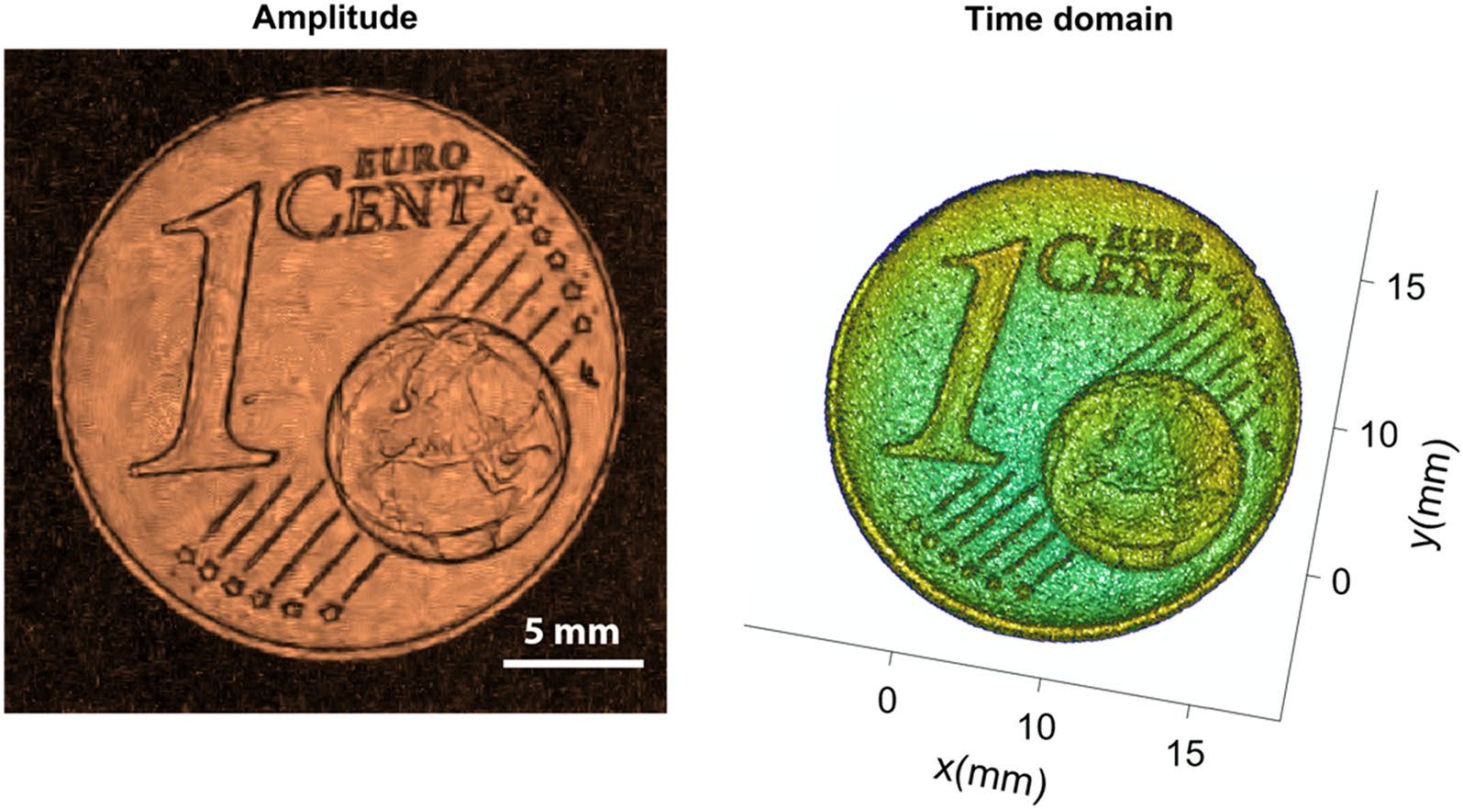
The figure displays the BM4D-filtered data for low-amplitude signals with an amplitude of 0.25V_pp_. It represents both the amplitude image (left) and the corresponding time domain image (right) obtained after applying the BM4D filter.

The line profiles of each denoised image also verify the superiority of the BM4D filter in reducing noise. The proposed denoising algorithm consistently outperforms other conventional filters, resulting in the most accurate and noise-free images. Figures 8 and 9 display the final denoised output of the BM4D filter, presenting denoised amplitude and time domain signal plots for both 0.24 Vpp and 0.25 Vpp low amplitude signals. These figures reinforce the effectiveness and robustness of the proposed denoising algorithm in handling low-amplitude data and improving the overall image quality.

### Quantitative analysis of denoised images

During the evaluation of the filtered output, we encountered an issue where the noisy data and the ground truth data had slight translation and rotation deviations. This misalignment affected the accuracy of peak-signal-to-noise ratio (PSNR) and structural similarity index measure (SSIM) evaluations, making it challenging to obtain reliable results.

To overcome this challenge, we utilized an image registration technique implemented through MATLAB. This technique allowed us to align the noisy data with the ground truth data accurately, correcting for any translation and rotation deviations. As a result, we obtained more precise measurements of PSNR and SSIM, providing a more reliable assessment of the denoising performance achieved by the various filters. With the corrected evaluations, we were able to confirm that the BM4D filter yielded the best denoising results, significantly reducing noise while preserving the image's structural details. The line profile analysis further supported the effectiveness of the BM4D filter in reducing total noise in the image. Overall, the application of the image registration technique provided a more comprehensive and accurate evaluation of the denoising algorithms' performance.

Determining the similarity between the ground truth (reference image) and filtered images is an essential component for calculating the peak signal-to-noise ratio (PSNR) and structural similarity index measure (SSIM)^39^. A SAM image with appropriate amplitude is chosen as the reference image, while a low amplitude SAM image is used as the sensed image. Once these images have been registered, they are compared based on their content. After processing the low-amplitude data using various denoising filters, it was necessary to evaluate the quality of the output obtained. In this regard, two commonly used measures, namely peak signal-to-noise ratio (PSNR) and structural similarity index measure (SSIM), were employed to quantitatively assess the performance of the denoising filters.

The PSNR is a commonly used metric to evaluate the quality of a denoised signal by computing the ratio of the peak signal power to the mean squared error (MSE) between the original and the denoised signal. Higher PSNR values indicate better performance of the denoising filter in preserving the signal quality. Similarly, the SSIM measures the structural similarity between the original and the denoised signal by comparing their luminance, contrast, and structural information. SSIM values range from 0 to 1, where a value of 1 indicates a perfect match between the original and denoised signals. The resulting PSNR and SSIM values obtained from the denoised signals of low amplitude data (0.24V_pp_ and 0.25V_pp_) were tabulated in Tables 1 and 2 to facilitate a direct comparison of the performance of each denoising filter. This evaluation process ensures the effectiveness of the proposed method for improving the quality of noisy signals.

**Table 1:** The table represents the peak signal-to-noise ratio (PSNR) and structural similarity index measure (SSIM) value obtained after image registration of the resultant data compared to the ground truth data, here the noi sy data used is the low amplitude 0.24V_pp_.

Filters	PSNR	SSIM
Gaussian	20.20	0.21
Median	20.09	0.20
Wiener	20.57	0.16
BM3D	20.22	0.24
BM4D	26.96	0.58
BM4D & BM3D	31.88	0.87

**Table 2:** The table represents the peak signal-to-noise ratio (PSNR) and structural similarity index measure (SSIM) value obtained after image registration of the resultant data compared to the ground truth data, here the noisy data used is the low amplitude 0.25V_pp_.

Filters	PSNR	SSIM
Gaussian	32.11	0.80
Median	32.31	0.82
Wiener	31.21	0.78
BM3D	31.96	0.82
BM4D	36.55	0.91
BM4D & BM3D	39.78	0.97

The quantitative analysis based on the PSNR and SSIM values shows that the combined BM4D and BM3D filter shows the best result which has the highest PSNR and SSIM values followed by the BM4D filter (Tables 1 and 2). Other applied conventional filter has lower performance compared to the proposed algorithm. We conducted a comprehensive analysis on the images, varying the amplitude (0.24 V and 0.25 V) of the input signal. This approach was adopted to showcase the robustness and effectiveness of the proposed denoising method. The results of this analysis are clearly presented in the tables (Tables 1 and 2), providing valuable insights into the performance of our method under different signal amplitudes. This in-depth analysis proves that our proposed algorithm is best suited to denoise the noisy data acquired from the SAM technique.

## Conclusion

In this paper, we have demonstrated a 4D block-matching filter can be used to denoise the scanning acoustic microscopic volumetric signals. Here we demonestrated restoring the noisy data obtained at the low-amplitude signal scans or noisy images with a low signal-to-noise ratio. The low amplitude signal data scanned are 0.25V_pp_ and 0.24V_pp_. We have compared it with various conventional denoising filters such as the Gaussian filter, Median filter and Wiener filter and compared the image with our proposed 4D & 3D block matching filter. From the visual inspection of the image and pondering over the peak signal-to-noise ratio (PSNR) and structural similarity index measure (SSIM) values obtained, it is evident that the proposed block match filter performed better than the compared conventional denoising filters when applied over time domain signals. The proposed block matching filter would be a good option in image denoising where the signal-to-noise ratio is poor, like in photoacoustic imaging (Supplementary Information).

### Supplementary Information

Supplementary Information.

## Data Availability

The authors declare the availability of the data and codes used in the research to obtain the results reported in the manuscript upon reasonable request from the corresponding author.
