# Supplementary material for: Image denoising in acoustic microscopy using block-matching and 4D filter
Source: Sci Rep. 2023 Aug 14;13:13212. doi: 10.1038/s41598-023-40301-7 (PMC10425453; doi:10.1038/s41598-023-40301-7)
Supplement: Supplementary file 1 — Supplementary Information. [file 41598_2023_40301_MOESM1_ESM.docx]

**Supplementary section**

**Image registration:**

Image registration refers to the process of aligning two or more images of the same event from different perspectives or captured by different sensor (1, 2). The captured images are geometrically aligned with respect to a reference image. Figure 1 represents the image registration process adopted in this manuscript.

**Figure 1:** Illustrates a schematic diagram that represents the four steps involved in image registration.

Image registration is a valuable tool in applications where images are acquired through various methods, such as: a) capturing images of the same scene from different viewpoints, b) capturing images of the same scene at different times, c) capturing images of the same scene using different sensors, and d) registering a scene to a model. However, due to the variability of images and various types of degradation, there is no universal method for image registration that is practical for all scenarios. When performing image registration, it is important to consider factors such as geometric deformation among the images, required accuracy, and noise content. However, most registration methods typically involve the following four steps:

**i)** Feature detection - This step involves detecting salient and distinctive features in the images, which can be done manually or automatically.

**ii)** Feature matching - The next step involves establishing a spatial relationship between the detected features in the sensed image and those in the reference image.

**iii)** Transform model estimation - Once the spatial relationship has been established, the parameters of the mapping functions are computed to create a transform model.

**iv)** Image resampling and transformation - The final step involves using the mapping functions to transform the sensed image. Interpolation techniques are used to calculate image values in coordinate locations where information may be missing after the transformation.

***Qualitative analysis of denoised images:***

The qualitative analysis of denoised images reveals the effectiveness of our denoising method. The denoised images exhibit improved visual quality with reduced noise compared to the noisy images. The method successfully preserves important details and structures in the images while effectively removing unwanted noise artifacts. Overall, the qualitative analysis confirms the success of our denoising approach in enhancing the clarity and quality of the images. In this study, we have included an additional sample with the amplitude of the excitation signal set to the minimum value (0.21V). This allows us to explore the performance of the denoising filters under more challenging conditions and assess their ability to handle low-amplitude data effectively. By considering this new sample, we can gain valuable insights into the filters' performance across a broader range of signal amplitudes and further validate their effectiveness in denoising acoustic data.


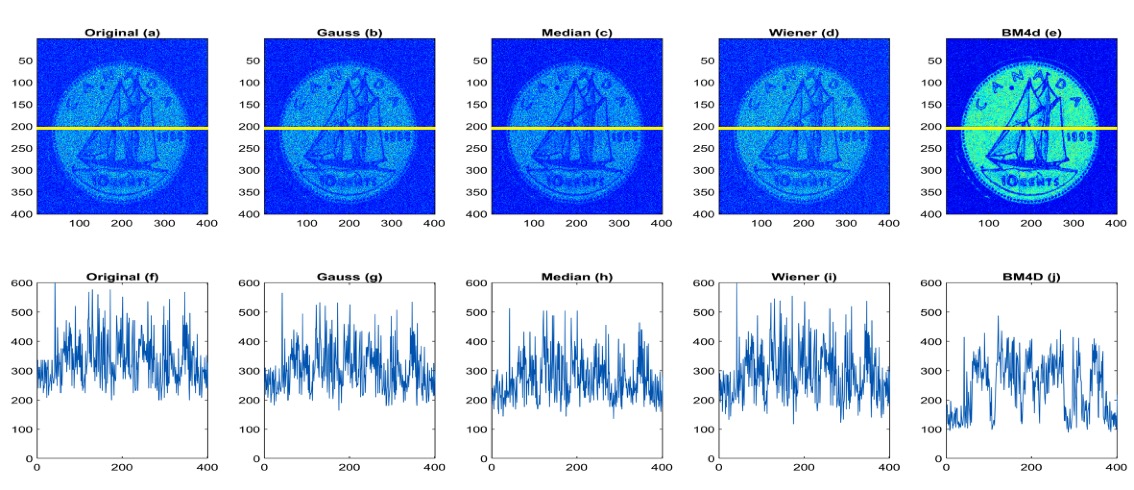


Figure 2: The figure demonstrates the application of various denoising filters, such as Gaussian, Median, Wiener, and BM4D filters, on low-amplitude signal data with an amplitude of 0.21Vpp. Each filter's denoised outputs are presented, enabling a comprehensive comparison of their noise reduction capabilities and their ability to enhance signal clarity. Through this analysis, we can evaluate the effectiveness of these filters in handling denoising for signals with low amplitudes.

Figure 2 displays the outcomes of various denoising filters applied to low-amplitude signal data with an amplitude of 0.21Vpp, aiming to evaluate their efficacy in enhancing output quality. The figure includes an amplitude image for each filter along with the corresponding line profile at Y = 201 (indicated by the yellow line). The denoising filters presented are: (a) the noisy data, (b) after applying a Gaussian filter, (c) after applying a median filter, (d) amplitude image after applying a Wiener filter, and (e) amplitude image after applying a 4D block matching filter to the time domain signals of the noisy data. This comprehensive analysis allows for a comparative assessment of each filter's noise reduction and signal enhancement capabilities.


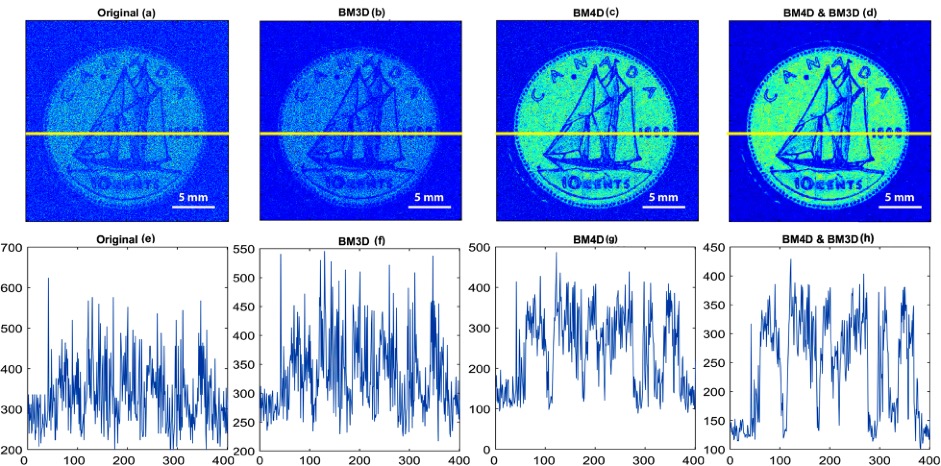


Figure 3: In Figure 3, we present the results of applying three denoising filters - BM3D, BM4D, and a combined approach of BM4D and BM3D - to low-amplitude signal data with an amplitude of 0.21Vpp. The denoised outputs obtained from each filter are shown, enabling a comparison of their noise reduction capabilities and their ability to enhance the clarity of the signal. This comprehensive analysis allows for a thorough evaluation of the effectiveness of these filters in handling denoising for low-amplitude signals.

Figure 3 presents a comprehensive evaluation of the performance of 4D and 3D block-matching filters in enhancing the quality of low-amplitude signal data with an amplitude of 0.21Vpp. The figure includes (a) the original noisy data, (b) the amplitude image after applying a 4D block-matching (BM4D) filter to the time domain signals of the noisy data, and (c) the amplitude image after applying a 3D block-matching (BM3D) filter to the image obtained from filtering the data with the BM4D algorithm. Additionally, the line profile at Y = 201 (indicated by the yellow line) for each image is included in the figure.

Qualitatively assessing the images in Figure 3, we observe that the BM4D filter effectively removes noise from the original image. Furthermore, applying the BM3D filter to the denoised image obtained from the BM4D algorithm significantly reduces noise while preserving the image structure and overall quality. This assessment is further supported by the line profile, which provides valuable insights into the overall performance of the combined BM4D and BM3D filter.

In this study, we specifically worked with a particular sample for which we did not acquire any ground truth image. Despite the absence of ground truth images, we were able to draw meaningful conclusions from the results obtained using various denoising filters. Unlike some deep learning-based approaches that heavily rely on ground truth images for training and evaluation, our proposed method does not necessitate ground truth images. The above-demonstrated images clearly illustrate the effectiveness of the denoising filters in enhancing the quality of the low-amplitude signal data. We were able to qualitatively assess the denoised outputs and draw meaningful insights from the visual analysis. However, due to the absence of ground truth images, we could not perform a quantitative analysis using metrics such as Peak Signal-to-Noise Ratio (PSNR) or Structural Similarity Index Measure (SSIM). Nevertheless, our results demonstrate the potential of the proposed denoising method in effectively reducing noise and improving the overall quality of the images, even in the absence of ground truth data.

***References***

1. B. Zitova and J. Flusser, "Image registration methods: a survey," *Image and vision computing,* vol. 21, no. 11, pp. 977-1000, 2003.

2. L. G. Brown, "A survey of image registration techniques," *ACM computing surveys (CSUR),* vol. 24, no. 4, pp. 325-376, 1992.
